# Immunological and short-term brain volume changes in relapsing forms of multiple sclerosis treated with interferon beta-1a subcutaneously three times weekly: an open-label two-arm trial

**DOI:** 10.1186/s12883-015-0488-9

**Published:** 2015-11-11

**Authors:** Michael G. Dwyer, Robert Zivadinov, Yazhong Tao, Xin Zhang, Cheryl Kennedy, Niels Bergsland, Deepa P. Ramasamy, Jackie Durfee, David Hojnacki, Bianca Weinstock-Guttman, Brooke Hayward, Fernando Dangond, Silva Markovic-Plese

**Affiliations:** Department of Neurology, Buffalo Neuroimaging Analysis Center, State University of New York at Buffalo, 100 High St, Buffalo, NY 14203 USA; Department of Biomedical Informatics, State University of New York at Buffalo, 100 High St, Buffalo, NY 14203 USA; Department of Neurology, State University of New York at Buffalo, 100 High St, Buffalo, NY 14203 USA; Department of Neurology, Microbiology and Immunology, University of North Carolina at Chapel Hill, 125 Mason Farm Rd., 6109D Neuroscience Research Bldg, CB #7125, Chapel Hill, NC 27599 USA; EMD Serono, Inc., One Technology Pl, Rockland, MA 02370 USA

**Keywords:** Multiple sclerosis, Pseudoatrophy, Interferon, Brain volumetry, Magnetic resonance imaging

## Abstract

**Background:**

Brain volume atrophy is observed in relapsing–remitting multiple sclerosis (RRMS).

**Methods:**

Brain volume changes were evaluated in 23 patients with RRMS treated with interferon β-1a 44 μg given subcutaneously (SC) three times a week (tiw) and 15 healthy controls. Percentages of whole brain and tissue-specific volume change were measured from baseline (0 months) to 3 months, from 3 to 6 months, and from baseline to 6 months using SIENAX Multi Time Point (SX-MTP) algorithms. Immunological status of patients was also determined and correlations between subsets of T cells and changes in brain volume were assessed.

**Results:**

Interferon β-1a 44 μg SC tiw in 23 patients with RRMS resulted in significant reductions in whole brain and gray matter tissue volume early in the treatment course (baseline to 3 months; mean change; –0.95 %; *P* = 0.030, –1.52 %; *P* = 0.004, respectively), suggesting a short-term treatment-induced pseudoatrophy effect. From baseline to 6 months, there were significant correlations observed between decreased T- cell expression of IL-17 F and decreased whole brain and brain tissue-specific volume.

**Conclusions:**

These findings are consistent with the interpretation of the pseudoatrophy effect as resolution of inflammation following treatment initiation with interferon β-1a 44 μg SC tiw, rather than disease-related tissue loss.

**Trial registration:**

ClinicalTrials.gov; NCT01085318

## Background

Relapsing–remitting multiple sclerosis (RRMS) is associated with ongoing loss in brain volume (atrophy) [1], and this loss contributes to irreversible neurologic impairment and cognitive decline [1–4]. Several clinical trials of disease-modifying drugs (DMDs) include some form of brain atrophy measurement as an endpoint [5–10] or in subgroup or post hoc analyses [1, 11–17]. Although treatment has been shown to significantly reduce the rate of brain volume loss compared with no treatment, this rate remains substantially higher than that seen in healthy controls [18]. Somewhat paradoxically, despite a long-term reduction in atrophy rate, initiation of anti-inflammatory therapy itself has been associated with brief but detectable reductions in brain volume – an effect termed pseudoatrophy [19]. This pseudoatrophy effect is thought to reflect hydrodynamic changes related to resolution of inflammation, edema, and cellular infiltration rather than the loss of actual brain tissue [19, 20].

Because both destructive tissue atrophy and these beneficial/benign pseudoatrophy changes may result in similar observations of brain volume reduction and confound efficacy measurements [21], it is critical to disentangle the two to understand the true impact of DMDs. Some initial work was done by looking at atrophy rates over different study time periods [9], and showed that the effect was predominantly driven by loss in white-matter (WM) volume; this loss was greater with higher initial inflammatory activity as measured by gadolinium-enhancing lesions. However, a full understanding remains elusive owing to the relative non-specificity of in vivo measurement methods. One important complementary approach might be to evaluate brain volume changes in combination with key immunological markers.

Response to DMDs and the occurrence of pseudoatrophy may be influenced by the underlying immunological status of patients prior to and during treatment. A priori, an association in patients with greater reduction in inflammatory markers and a greater amount of pseudoatrophy over the same time period would be expected. Chronic inflammation in MS is driven by production of pro-inflammatory cytokines, such as interferon gamma (IFN γ) and interleukin (IL)-17 by CD4^+^ T cells [22], which promote the disruption of blood–brain barrier integrity, allowing egress of activated T cells into the central nervous system (CNS), targeting of CNS-resident cells and resulting in demyelination [23]. The presence of CD4^+^ T cells and an abundance of CD8^+^ T cells in MS lesions is well documented [24, 25].

The efficacy of IFN beta-1a given subcutaneously three times a week (IFN β-1a SC tiw) for the treatment of RRMS has been established [25–28]. However, its effect on early changes in brain volume and its relationship to changes in inflammatory markers have not been investigated previously.

Advanced imaging techniques are able to detect whole brain atrophy via change analysis directly between scans rather than through the more error-prone post hoc comparisons of cross-sectional data of whole brain volume measurement over time [29]. Direct measurement of whole brain volume change is possible with approaches such as the Structural Image Evaluation using Normalization of Atrophy (SIENA) method [30]. This technique measures the percent of whole brain volume change between scans of the same subject acquired at specific time points during the study (eg, at baseline and following treatment). However, since SIENA does not allow for discrimination of tissue-specific atrophy, it is not possible to evaluate the relative contributions of gray matter (GM) and WM to whole brain atrophy in MS. As it is important to gain a more comprehensive insight into the relative contributions of these two compartments to MS pathophysiology, this study used the recently described SIENAX Multi Time Point (SX-MTP) analysis algorithm [29] to allow for the direct and sensitive measurement of tissue-specific brain volume changes.

### Study objectives

The Rebif Advanced MRI and Immunology Pilot Trial (NCT01085318) [31] was a two-arm, open-label, 6-month trial conducted to evaluate remyelination/demyelination, brain volume, and iron deposition in the CNS, as well as the immunological status of 23 patients with RRMS treated with IFN β-1a 44 μg SC tiw compared with 15 healthy controls (HCs). The objective of the present study was to measure changes in global (whole brain) and tissue-specific (GM and WM) percent brain volume in patients following initiation of treatment with IFN β-1a SC tiw. Comparisons with changes in HCs were carried out, as were investigations of the correlations between potential immunological biomarkers, gadolinium (Gd)-enhancing lesions, and short-term brain volume changes following treatment initiation.

## Methods

Eligible patients were 18–65 years of age, with a diagnosis of RRMS according to the 2010 revised McDonald criteria [32]. Patients received IFN β-1a SC tiw titrated to 44 μg for 6 months. For all participants, conventional magnetic resonance imaging (MRI) exams of the brain were performed on a 3 T GE Signa Excite 12.0 scanner at baseline and at 3- and 6-month follow-up visits.

### Ethics, consent, and permissions

The trial was conducted in accordance with the International Conference on Harmonisation guidelines for Good Clinical Practice and applicable local regulations, as well as the Declaration of Helsinki. Written informed consent was given by patients before participation and the protocol was approved by the Institutional Review Board at the University at Buffalo Health Sciences.

### Brain volume analysis

Percent of whole brain volume change was measured from baseline (0 months) to 3 months, from 3 to 6 months, and from baseline to 6 months using SIENA analysis algorithms [30]. Serial scans were co-registered and relative edge motions (measuring expansion or contraction of the brain) were detected between scans and used to extrapolate the percent change in brain volume. SIENA measurements were improved by application of an in-house developed inpainting technique, nonuniformity correction [33], and intensity standardization [34]. Changes in GM and WM volumes were measured during the same study periods described above using the SX-MTP analysis algorithms on the inpainted, corrected, and standardized images. For GM/WM segmentation, 4D accuracy was improved through MTP modification via longitudinal regularization of a hidden Markov random field model.

### Immunological markers of inflammation: screening

For the immunological marker analysis, CD4^+^ and CD8^+^ T-cell expression of an array of immunological markers with known immunomodulatory properties was quantified at baseline and at 6 months. Blood samples were collected from patients. Peripheral blood mononuclear cells (PBMCs) were placed in serum-free medium and stimulated with phorbol myristate acetate (50 ng/mL) and Ionomycin (500 ng/mL; Sigma, St. Louis, MO) for 2 h and brefeldin A (1:1000 dilution; eBioscience, San Diego, CA) for an additional 3 h for the intracellular cytokine staining. PBMCs were fixed, permeabilized, and stained with fluorescein-conjugated antibodies against IFN γ, IL-4, IL-17A, IL-21, IL-22 (eBioscience, San Diego, CA), IL-17 F, brain-derived neurotrophic factor (BDNF; R&D Systems, Minneapolis, MN), IL-10, CD4, and CD8 (BD Biosciences, San Jose, CA). For nerve growth factor (NGF) staining, the cells were stained with primary antibody against NGF (Epitomics, Cambridge, MA), followed by staining with fluorescein isothiocyanate-conjugated secondary antibody (BD Bioscience). The percent of CD4^+^ and CD8^+^ T cells expressing each marker of interest was determined using a BD FACSCalibur™ flow cytometer and CellQuest software (BD Biosciences) and gated by size and granularity.

### Statistics

All statistical comparisons were planned in advance of data collection. The Wilcoxon signed-rank test was used to test for within-group differences in percent of brain volume change (whole brain, GM, and WM) at each of the study periods (from baseline to 3 months, from 3 months to 6 months, and from baseline to 6 months). The Wilcoxon rank-sum test was used to test for differences in percent of whole brain volume change, GM, and WM between patients and HCs during each study period. Correction for multiple comparisons by the Holm-Bonferroni method was applied. Spearman’s rank correlation was used to test the correlations between immunological parameters, Gd-enhancing lesions at baseline, and percent change in brain volume and GM and WM over 3 and 6 months of treatment. No corrections for multiple comparisons were made to the correlation results.

## Results

All 15 HCs and 21/23 patients completed the study; one patient was lost to follow-up and one patient discontinued owing to the investigator’s decision. The mean age (standard deviation [SD]) of the enrolled patients was 39.9 (10.17) years and 61 % (14/23) of the patients were female. Baseline characteristics were similar between patients and HCs; patient baseline characteristics were described previously in the primary study [31].

### Whole brain volume analysis

**Fig. 1:**
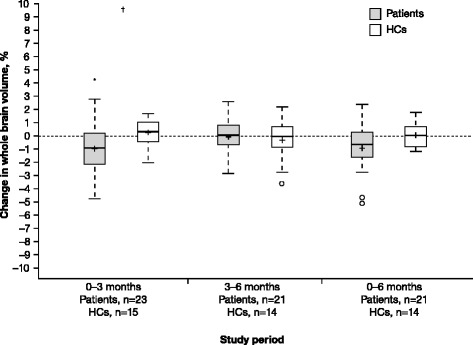
Brain volume changes in RRMS patients vs HCs. Percent change in whole brain volume in RRMS patients treated with IFN β-1a SC tiw and in HCs was measured. No *P*-values remained significant after adjustment for multiple comparisons. *P < 0.05, within-group difference from zero (Wilcoxon signed-rank test). ^†^ *P* < 0.05, patients versus HCs (Wilcoxon rank-sum test). In the box plots, the bold line represents the median; the boxes represent the middle 50 % of data; the top and bottom of the box represent the third and first quartiles; the open circles are outliers. The whisker lines above and below the boxes represent the largest and smallest values that are not considered to be outliers. Means are denoted by a ‘+’ sign. *HC* healthy control; *IFN* interferon; *RRMS* relapsing–remitting multiple sclerosis; *SC* subcutaneously; *tiw* three times weekly

Whole brain volume analysis is shown in Table 1 and Fig. 1. In patients, the mean (SD) change from baseline to 3 months in brain volume was –0.95 % (1.71; *P* = 0.030 for difference from baseline; *P* = 0.090 after adjusting for multiple comparisons), –0.08 % (1.36; *P* = 0.960) from 3 to 6 months, and –0.91 % (1.88; *P* = 0.051) from baseline to 6 months. For comparison, in HCs the mean change from baseline to 3 months in brain volume was 0.24 % (1.07; *P* = 0.359), –0.32 % (1.50; *P* = 0.626) from 3 to 6 months, and 0.01 % (1.89; *P* = 0.952) from baseline to 6 months.

**Table 1:** Brain volume changes in RRMS patients treated with IFN β-1a SC tiw and HCs over time

	Baseline to 12 weeks		12 to 24 weeks		Baseline to 24 weeks	
	Patients	HCs	Patients	HCs	Patients	HCs
n (missing^a^)	23 (0)	15 (0)	21 (2)	14 (1)	21 (2)	14 (1)
Percent change in whole brain (%)						
Mean (SD)	–0.95 (1.712)	0.24 (1.068)	–0.08 (1.358)	–0.32 (1.500)	–0.91 (1.880)	0.01 (0.885)
Median	–0.90	0.33	0.07	–0.02	–0.63	0.05
Min, max	–4.7, 2.8	–2.0, 1.7	–2.8, 2.6	–3.6, 2.2	–5.1, 2.4	–1.2, 1.8
*P* value^b^	0.030	0.359	0.960	0.626	0.051	0.952
Adjusted *P-*value^b,c^	NS	NS	NS	NS	NS	NS
*P-*value^d^	0.015		0.702		0.071	
Adjusted *P-*value^c,d^	NS		NS		NS	
Adjusted *P-*value^c,e^	NS/NS					
Percent change in gray matter (%)						
Mean (SD)	–1.52 (2.406)	0.01 (2.415)	–0.46 (3.109)	–0.60 (2.189)	–1.66 (1.836)	–0.51 (2.472)
Median	–1.89	0.80	–1.20	–0.72	–1.24	–1.31
Min, max	–6.3, 2.3	–3.7, 3.7	–6.3, 9.4	–4.4, 2.5	–5.9, 1.7	–4.5, 5.1
*P-*value^b^	0.004	0.934	0.176	0.426	<0.001	0.268
Adjusted *P-*value^b,c^	0.0286	NS	NS	NS	0.0016	NS
*P-*value^d^	0.093		0.778		0.342	
Adjusted *P-*value^c,d^	NS		NS		NS	
Adjusted *P-*value^c,e^	NS/NS					
Percent change in white matter (%)						
Mean (SD)	–0.41 (2.159)	0.51 (1.940)	0.30 (2.991)	–0.03 (1.985)	–0.21 (3.202)	0.58 (2.461)
Median	–0.63	0.69	0.93	0.50	0.08	0.95
Min, max	–5.4, 3.5	–5.1, 3.0	–6.5, 7.4	–3.4, 2.7	–5.9, 8.4	–4.5, 3.6
*P-*value^b^	0.390	0.107	0.626	0.952	0.933	0.463
Adjusted *P-*value^b,c^	NS	NS	NS	NS	NS	NS
*P-*value^d^	0.093		0.630		0.309	
Adjusted *P-*value^c,d^	NS		NS		NS	
Adjusted *P-*value^c,e^	NS/NS					

^a^Failed analysis or missing MRI at Week 24

^b^For the difference from zero within groups from the Wilcoxon signed-rank test

^c^Adjusted for multiple comparisons with the Holm-Bonferroni correction; NS denotes adjusted *P-*values ≥ 0.05

^d^For the difference between IFN β-1a SC tiw and HCs from the Wilcoxon rank-sum test

^e^For the difference between the first 12 weeks and the second 12 weeks within groups from the Wilcoxon signed-rank test (*P-*value for patients/HCs)

*GM* gray matter, *HC* healthy control; max: maximum; min: minimum, *NS* non-significant, *SD* standard deviation

### GM volume analysis

**Fig. 2:**
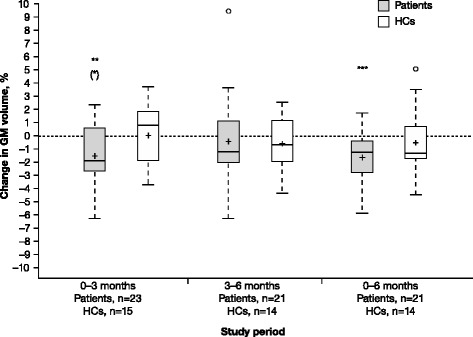
Gray matter volume changes in RRMS patients and HCs. Percent change in GM volume in RRMS patients treated with IFN β-1a SC tiw and in HCs was assessed. *P*-values were for the difference from zero within groups from the Wilcoxon signed-rank test. *P*-values in parentheses are those remaining significant after Holm–Bonferroni correction for multiple comparisons. *P < 0.05. **P < 0.01. ***P < 0.001. In the box plots, the bold line represents the median; the boxes represent the middle 50 % of data; the top and bottom of the box represent the third and first quartiles; the open circles are outliers. The whisker lines above and below the boxes represent the largest and smallest values that are not considered to be outliers. Means are denoted by a ‘+’ sign. *GM* gray matter; *HC* healthy control; *IFN* interferon; *RRMS* relapsing–remitting multiple sclerosis; *SC* subcutaneously; *tiw* three times weekly

GM volume analysis is shown in Table 1 and Fig. 2. The pattern for GM was similar to that of whole brain. In patients, the mean (SD) change from baseline to 3 months in GM volume was –1.52 % (2.41; *P* = 0.004 and after adjusting for multiple comparisons, *P* = 0.029), –0.46 % (3.11; *P* = 0.176) from 3 to 6 months, and –1.66 % (1.84; *P* < 0.001 and after adjusting for multiple comparisons, *P* = 0.002) from baseline to 6 months. There were no significant differences observed for the HCs: mean change from baseline to 3 months was 0.01 % (SD 2.42; *P* = 0.934), –0.60 % (2.19; *P* = 0.426) from 3 to 6 months, and –0.51 % (2.47; *P* = 0.268) from baseline to 6 months.

### WM volume analysis

**Fig. 3:**
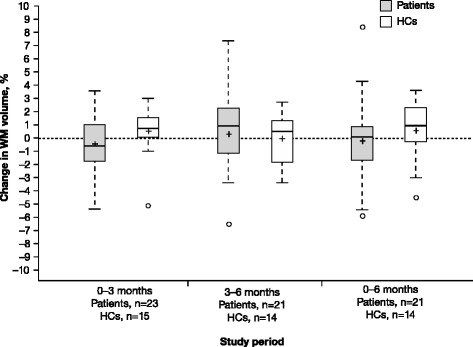
White matter volume changes in RRMS and HCs. Percent change in WM volume in RRMS patients treated with IFN β-1a SC tiw and in HCs was measured. Changes in WM volume were not significant at any timepoint. In the box plots, the bold line represents the median; the boxes represent the middle 50 % of data; the top and bottom of the box represent the third and first quartiles; the open circles are outliers. The whisker lines above and below the boxes represent the largest and smallest values that are not considered to be outliers. Means are denoted by a ‘+’ sign. *HC* healthy control; *IFN* interferon; *RRMS* relapsing–remitting multiple sclerosis; *SC* subcutaneously; *tiw* three times weekly; *WM* white matter

WM volume analysis is shown in Table 1 and Fig. 3. Changes in WM tissue volume of patients were not significant at any timepoint. In patients, the mean (SD) change from baseline to 3 months in WM volume was –0.41 % (2.16; *P* = 0.390), 0.30 % (2.99; *P* = 0.626) from 3 to 6 months, and –0.21 % (3.20; *P* = 0.933) from baseline to 6 months. In HCs, the mean (SD) change from baseline to 3 months in WM volume was 0.51 % (1.94; *P* = 0.107), –0.03 % (1.96; *P* = 0.952) from 3 to 6 months, and 0.58 % (2.46; *P* = 0.463) from baseline to 6 months.

### Correlations between changes in whole brain, GM, and WM, baseline Gd-enhancing lesions, and immunological markers following treatment

Only 8 of the patients with RRMS had any Gd-enhancing lesions at baseline, and there were no significant correlations between the number or volume of Gd-enhancing lesions at baseline and the changes in whole brain, GM, or WM volumes.

**Fig. 4:**
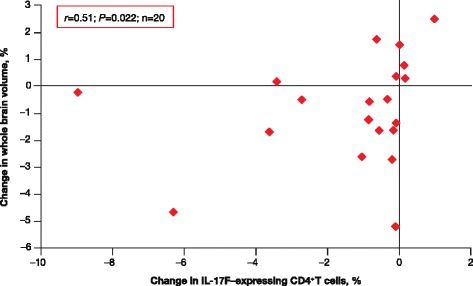
IL-17 F–expressing CD4+ T cells and brain volume changes in RRMS patients and HCs. Decreased percentage of IL-17 F–expressing CD4+ T cells from baseline to 6 months correlated with decreased whole brain volume from baseline to 6 months in RRMS patients treated with IFN β-1a SC tiw. *HC* healthy control; *IFN* interferon; *IL* interleukin; *RRMS* relapsing–remitting multiple sclerosis; *SC* subcutaneously; *tiw* three times weekly

There were a number of significant correlations between whole brain and brain tissue-specific volumes and immunological markers following 6 months of treatment with IFN β-1a SC. Decreased percentage of IL-17 F-expressing CD4^+^ T cells from baseline to 6 months correlated significantly with decreasing whole brain volume over the same period (*r* = 0.51; *P* = 0.022; Fig. 4).

**Fig. 5:**
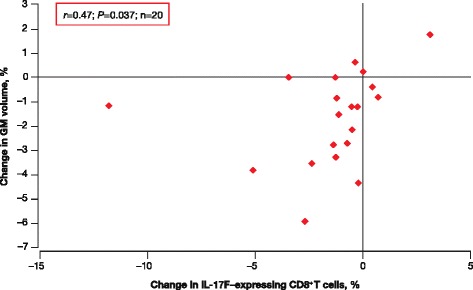
IL-17 F–expressing CD8^+^ T cells and GM volume changes in RRMS. Higher percentage of IL-17 F–expressing CD8^+^ T cells from baseline to 6 months correlated with smaller decreases in GM volume from baseline to 6 months in treated patients with RRMS. *GM* gray matter; *IL* interleukin; *RRMS* relapsing–remitting multiple sclerosis

**Fig. 6:**
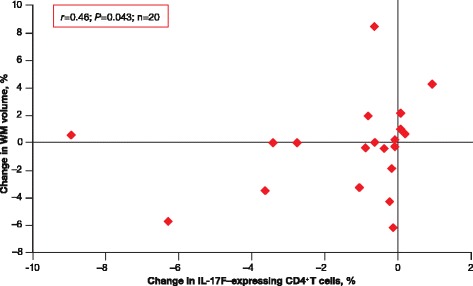
IL-17 F-expressing CD4^+^ T cells and WM volume changes in RRMS. Decreased percentage of IL-17 F-expressing CD4^+^ T cells from baseline to 6 months correlated with decreased WM volume from baseline to 6 months in treated patients with RRMS. *IL* interleukin; *RRMS* relapsing–remitting multiple sclerosis; *WM* white matter

Decreased percentage of IL-17 F-expressing CD8^+^ T cells from baseline to 6 months correlated with decreasing GM volume over the same period (*r* = 0.47; *P* = 0.037; Fig. 5). In addition, decreased percentage of IL-17 F-expressing CD4^+^ T cells from baseline to 6 months correlated with a decreasing WM volume (*r* = 0.46; *P* = 0.043; Fig. 6), suggestive of pseudoatrophy.

**Fig. 7:**
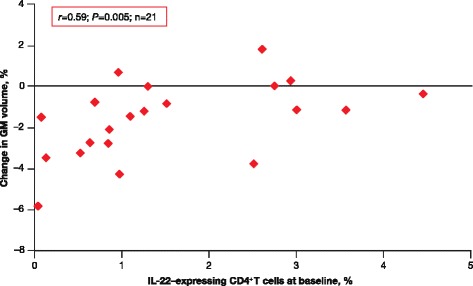
IL-22–expressing CD4^+^ T cells and GM volume changes in RRMS. Higher percentage of IL-22–expressing CD4^+^ T cells at baseline correlated with smaller decreases in GM volume from baseline to 6 months in treated patients with RRMS. *GM* gray matter; *IL* interleukin; *RRMS* relapsing–remitting multiple sclerosis

A higher percentage of IL-22–expressing CD4^+^ T cells at baseline correlated with smaller decreases in GM volume from baseline to 6 months (*r* = 0.59; *P* = 0.005; Fig. 7).

## Discussion

### Brain volume analysis

The aim of this study was to use advanced imaging techniques to measure the extent of global and tissue-specific brain volume changes, and to examine the involvement of IFN β-1a 44 μg SC tiw, in the pseudoatrophy phenomenon in patients with RRMS. A better understanding of the pathophysiological mechanisms in RRMS, including a differentiation of the dynamics of inflammation and demyelination leading to brain volume loss observed in MS, could aid in devising means for halting disease progression and for treatment monitoring and optimization. In particular, the early study period measurement from baseline to 3 months represents an opportunity to investigate acute treatment-associated pseudoatrophy following IFN β-1a 44 μg SC tiw therapy initiation. The investigation of brain volume loss in whole brain tissue, as well as in GM and WM, allowed for a greater insight into the specificity of tissue volume changes suggestive of pseudoatrophy. In whole brain and GM, the effect of IFN β-1a SC treatment was associated with a reduction in brain tissue volume; in both cases the majority of the reduction occurred shortly (within 3 months) following treatment initiation, in comparison with the 3–6-month period, a result that is highly consistent with an acute pseudoatrophy effect.

Treatment-associated pseudoatrophy has been thought previously to be more evident in WM than in GM [9, 35]. This was not observed in the present study, where decline in whole brain tissue volume loss appeared to be driven by GM tissue volume loss. GM loss was more pronounced in the first 3 months than the second 3 months, and only loss in the first 3 months was statistically significant. While pseudoatrophy owing to WM volume has been noted during treatment with natalizumab [9], the longer term brain tissue changes in RRMS and clinical measures (such as physical and cognitive disability) have been associated more with GM volume changes rather than loss of WM volume [36, 37].

This conflicting finding of a GM rather than a WM effect may be due to a number of factors. Given the small sample size, issues of statistical power cannot be ruled out – in fact, the observed but not statistically significant WM volume changes were in the expected direction and were lower from 0–3 months than from 3–6 months. Methodological effects should also be carefully considered. Although the SIENAX-MTP method employed has been shown to more accurately measure GM volume change than was previously possible, it was not compared head-to-head with SIENAX or SPM for this study. It is noteworthy, though, that cortical GM measurement is much more susceptible to partial volume errors owing to its thin and convoluted nature, and that previous studies using less sensitive analysis techniques failed to detect significant brain atrophy progression over a 3-month period in RRMS [38] or in an open-label study of 30 patients with RRMS treated with IFN β-1b [5].

If correct, though, these exploratory results merit speculation about the potential pathophysiological basis for the observed GM changes. Over the past decade, it has become clear that GM plays an enormous role in both the pathology and the clinical picture of multiple sclerosis [39]. What remains unclear is whether the involvement of GM is secondary to WM damage or a semi-independent process [40]. Of particular relevance to the current work is the inflammatory nature of GM pathology. Histopathological studies have found much lower inflammation in the GM than in the WM [41, 42]. However, biopsies have shown substantial inflammation [43, 44]. Notably, cortical inflammation appeared in patients with early RRMS, much like the cohort studies described here. It is also possible that different therapeutic mechanisms of DMDs may affect any pseudoatrophy of specific tissue compartments differently.

### Correlations between brain tissue volume, Gd-enhancing lesions, and immunological markers of inflammation

Investigating correlations between brain volume and pro- and anti-inflammatory markers has provided some new insight into the proposed mechanisms of action of IFN β-1a SC tiw. The significant correlations between the decreased percentage of pro-inflammatory IL-17 F–expressing CD4^+^ or CD8^+^ T cells and reductions in whole brain, GM, and WM tissue volumes are supportive of an early anti-inflammatory therapeutic effect for IFN β-1a SC tiw.

The correlation between higher baseline percentage of IL-22–expressing CD4^+^ T cells and less GM tissue atrophy from baseline to 6 months in patients is also of potential interest. Although interpretation of cross-sectional markers should be undertaken with care, it is possible that higher baseline IL-22 levels may be indicative of a different pseudoatrophy response – either via less resolvable baseline inflammatory-related volumetric changes or via differences in treatment response.

The comprehensive battery of immunological markers, including BDNF, was used to screen for correlations with brain tissue volume changes. Beyond IL-17 F and IL-22, no further significant correlations were observed, despite previous reports that BDNF secretion was associated with inflammatory activity in MS lesions in the brain and with higher WM tissue volume [45].

We did not detect a relationship between brain volume and the resolution of Gd-enhancing lesions, likely due to the high proportion of patients with no Gd-enhancing lesions at baseline.

### Implications for future investigations of brain volume changes

Our findings imply the need for caution when planning the timing of using MRI techniques to measure disease progression in terms of brain volume. They suggest that the baseline for MRI measures of brain volume in patients with RRMS with active disease embarking on DMD therapy should start only after several months of therapy, once the pseudoatrophy effect has run its course, rather than at the beginning of therapy, indicating a need for additional MRI measurements. Based on these results, the pseudoatrophy effect may account for a 1 % or more change in volume. Although seemingly small in an absolute sense, the yearly percent brain volume change in MS may be as low as 0.5 % (and 0.1 % in HCs). Thus, early pseudoatrophy over 3 months may produce volume changes commensurate with 2 years of MS disease effect, and completely mask treatment effects in clinical trials of that length. This is highly relevant, as such trials are becoming more common.

It is even conceivable that in the future more accurate MRI and analysis techniques might lead to individual patient care decisions based on rates of brain atrophy in addition to the conventionally used lesion burden. Any such initiatives will need to carefully account for the pseudoatrophy effect in order to be useful.

### Limitations

With the exception of GM tissue volume change from baseline to 3 months, the statistical significance of other associations observed was lost following adjustment for multiple comparisons. This may be as a result of this being a pilot study with a relatively small sample size, as well as the large number of exploratory comparisons that underwent correction. It is also possible that the improved SIENAX-MTP algorithm may be more powerful to detect real GM change than WM change [29]. Therefore, the lack of WM changes detected should be interpreted with caution.

The additional measurement of immunology marker changes from baseline to 3 months were also not feasible for this study, but would have enabled the same timepoint comparison with the brain volume analysis, contributing to the understanding of short-term changes in immunology alongside pseudoatrophy.

As GM and WM atrophy occurs with aging in normal controls [46–49], the use of the HC group in this study as a comparison with the treated patients provided a control for the normal aging processes but not for time course of brain volume changes in patients with RRMS. In future studies, an active comparator group may help to establish the extent to which treatment-related pseudoatrophy is affected by treatment with IFN β-1a SC in comparison with other DMDs. In addition, more frequent measurements over the first 6 months and extension of measurement periods would allow the chance to better delineate the time course over which pseudoatrophy occurs in order to be able to differentiate it from true atrophy.

## Conclusions

The results of this pilot study demonstrated a significant reduction in whole brain volume within 3 months following IFN β-1a 44 μg SC tiw treatment initiation, but not between 3 and 6 months, which is suggestive of treatment-induced pseudoatrophy. The observed reduction in pro-inflammatory cytokine IL-17 F expression by T cells correlating with whole brain and GM volume decreases is consistent with an anti-inflammatory effect of IFN β-1a SC normalizing brain volume to a pre-inflammatory level, and is consistent with the interpretation of the pseudoatrophy effect as resolution of inflammation rather than tissue destruction. Given these findings, attempts to measure treatment effects on atrophy should consider the use of an MRI baseline at least 3 months after treatment initiation.

## Abbreviations

BDNF: Brain-derived neurotrophic factor
CNS: Central nervous system
DMD: Disease-modifying drug
Gd: Gadolinium
GM: Gray matter
HC: Healthy control
IFN: Interferon
IL: Interleukin
MRI: Magnetic resonance imaging
NGF: Nerve growth factor
RRMS: Relapsing–remitting multiple sclerosis
SC: Subcutaneously
SD: Standard deviation
SIENA: Structural Image Evaluation using Normalization of Atrophy
SX-MTP: SIENAX Multi Time Point
tiw: three times weekly
WM: White matter
